# Activity of Cefiderocol and Comparators against Isolates from Cancer Patients

**DOI:** 10.1128/AAC.01955-19

**Published:** 2020-04-21

**Authors:** Kenneth V. I. Rolston, Baghat Gerges, Samuel Shelburne, Samuel L. Aitken, Issam Raad, Randall A. Prince

**Affiliations:** a Department of Infectious Diseases, Infection Control, and Employee Health, The University of Texas MD Anderson Cancer Center, Houston, Texas, USA; b Division of Pharmacy, The University of Texas MD Anderson Cancer Center, Houston, Texas, USA

**Keywords:** Gram-negative isolates, *Pseudomonas aeruginosa*, *Stenotrophomonas maltophilia*, cefiderocol

## Abstract

Cefiderocol inhibited 97.5% of 478 Gram-negative isolates from cancer patients at ≤4 mg/liter. It had potent activity against extended-spectrum β-lactamase-positive *Enterobacteriaceae*, carbapenem-resistant *Enterobacteriaceae* (CRE), and nonfermenting Gram-negative bacilli, including Pseudomonas aeruginosa, Stenotrophomonas maltophilia, and Acinetobacter species isolates. Amikacin, ceftazidime-avibactam, and meropenem had appreciable activity against non-CRE *Enterobacteriaceae*. No comparators were active against multidrug-resistant P. aeruginosa isolates. Only trimethoprim-sulfamethoxazole had appreciable activity against S. maltophilia isolates. Overall, cefiderocol was associated with the lowest level of resistance.

## Abstract (precis)

Cefiderocol inhibited 97.5% of 478 Gram-negative isolates from cancer patients at ≤4 mg/liter. It had potent activity against extended-spectrum β-lactamase-positive *Enterobacteriaceae*, carbapenem-resistant *Enterobacteriaceae* (CRE), and nonfermenting Gram-negative bacilli, including Pseudomonas aeruginosa, Stenotrophomonas maltophilia, and Acinetobacter species isolates. Amikacin, ceftazidime-avibactam, and meropenem had appreciable activity against non-CRE *Enterobacteriaceae*.

## TEXT

Gram-negative organisms (GNOs) are the predominant bacterial pathogens at many cancer centers, and many have developed resistance to commonly used antimicrobial agents (1–4). Cefiderocol (CFDC) is a new siderophore cephalosporin that has been reported to be more stable than other β-lactams against β-lactamases, including NDM-1 and KPC-3 carbapenemases (5–6). Its *in vitro* activity has been evaluated against GNOs from various sources, but data against organisms from cancer patients are rare (7). We evaluated its activity against GNOs isolated exclusively from cancer patients. All organisms tested were clinical isolates (2014 to 2017), with >90% from blood cultures. MICs were determined using a broth microdilution method (8). When available, CLSI or FDA breakpoints for susceptibility and resistance were used. Ten comparator agents were tested (Table 1). Trimethoprim-sulfamethoxazole was tested instead of aztreonam against Stenotrophomonas maltophilia only. Whole-genome sequencing was done on the 12 isolates that were nonsusceptible to CFDC (1 each, Escherichia coli, Pseudomonas aeruginosa, and *Citrobacter* spp.; 2 Klebsiella pneumoniae; 3 Acinetobacter spp.; and 4 *Enterobacter* spp.) (9).

**TABLE 1:** *In vitro* activity of cefiderocol and comparator agents against select clinical isolates

Isolate, type	Antimicrobial agent	MIC (mg/liter)		Susceptible (%)
		MIC_90_	Range	
ESBL positive				
E. coli (*n* = 52)	Cefiderocol	2	<0.03 to 4	100
	Ceftolozane-tazobactam	32	0.125 to >64	82
	Meropenem	0.06	<0.03 to 0.25	100
	Ceftazidime	>64	1 to >64	15
	Ceftazidime-avibactam	1	<0.03 to 4	100
	Colistin	1	1 to 4	NA
	Aztreonam	>32	<0.5 to >32	10
	Amikacin	16	<4 to 32	98
	Ciprofloxacin	>4	<0.25 to >4	8
	Cefepime	>16	<0.5 to >16	10
	Tigecycline	0.5	<0.25 to 2	100
K. pneumoniae (*n* = 37)	Cefiderocol	2	0.125 to >64	97
	Ceftolozane-tazobactam	32	0.25 to >64	62
	Meropenem	0.125	<0.03 to 4	97
	Ceftazidime	>64	4 to >64	0
	Ceftazidime-avibactam	0.5	<0.03 to >64	97
	Colistin	>8	1 to >8	NA
	Aztreonam	>32	8 to >32	0
	Amikacin	16	<4 to >64	92
	Ciprofloxacin	>4	<0.25 to >4	11
	Cefepime	>16	1 to >16	11
	Tigecycline	>4	<0.25 to >4	54
CRE (*n* = 40: 10 E. coli, 7 E. cloacae, and 23 K. pneumoniae)	Cefiderocol	4	0.06 to >64	95
	Ceftolozane-tazobactam	>64	0.5 to >64	18
	Meropenem	>64	<0.03 to >64	18
	Ceftazidime	>64	16 to >64	0
	Ceftazidime-avibactam	>64	0.06 to >64	78
	Colistin	>8	<0.5 to >8	NA
	Aztreonam	>32	16 to >32	0
	Amikacin	64	<4 to >64	73
	Ciprofloxacin	>4	<0.25 to >4	15
	Cefepime	>16	<0.5 to >16	68
	Tigecycline	4	<0.25 to >4	65
*Citrobacter* spp. (*n* = 20)	Cefiderocol	1	<0.03 to 8	95
	Ceftolozane-tazobactam	64	0.06 to >64	70
	Meropenem	0.25	<0.03 to 8	95
	Ceftazidime	>64	0.25 to >64	60
	Ceftazidime-avibactam	1	0.06 to 8	100
	Colistin	2	1 to >8	NA
	Aztreonam	>32	<0.5 to >32	55
	Amikacin	<4	<4	100
	Ciprofloxacin	>4	<0.25 to >4	70
	Cefepime	16	<0.5 to >16	80
	Tigecycline	2	<0.25 to 4	95
E. cloacae (*n* = 38)	Cefiderocol	4	<0.03 to > 64	90
	Ceftolozane-tazobactam	>64	0.06 to >64	55
	Meropenem	1	<0.03 to 64	90
	Ceftazidime	>64	0.125 to >64	95
	Ceftazidime-avibactam	4	0.125 to 16	95
	Colistin	>8	1 to > 8	NA
	Aztreonam	>32	<0.5 to >32	45
	Amikacin	8	<4 to 16	100
	Ciprofloxacin	>4	<0.25 to >4	63
	Cefepime	>16	<0.5 to >16	66
	Tigecycline	2	<0.25 to >4	90
*Serratia* spp. (*n* = 20)	Cefiderocol	0.5	<0.03 to 0.5	100
	Ceftolozane-tazobactam	0.5	0.25 to 1	100
	Meropenem	0.125	<0.03 to 0.125	100
	Ceftazidime	0.5	0.25 to 0.5	100
	Ceftazidime-avibactam	0.5	0.06 to 0.5	100
	Colistin	>8	>8	NA
	Aztreonam	<0.5	<0.5	100
	Amikacin	8	<4 to 8	100
	Ciprofloxacin	<0.25	<0.25 to 0.5	95
	Cefepime	<0.5	<0.5	100
	Tigecycline	2	1 to 2	100
Acinetobacter spp. (*n* = 20)	Cefiderocol	4	<0.03 to >64	90
	Ceftolozane-tazobactam	>64	<0.03 to >64	NA
	Meropenem	>64	<0.03 to >64	75
	Ceftazidime	>64	2 to >64	45
	Ceftazidime-avibactam	32	0.06 to >64	NA
	Colistin	2	1 to 2	100
	Aztreonam	32	8 to 32	NA
	Amikacin	16	<4 to >64	95
	Ciprofloxacin	>4	<0.25 to >4	70
	Cefepime	>16	<0.5 to >16	70
	Tigecycline	2	<0.25 to 4	NA
P. aeruginosa, MDR (*n* = 32)	Cefiderocol	1	<0.03 to > 64	97
	Ceftolozane-tazobactam	>64	0.5 to >64	66
	Meropenem	>64	0.5 to >64	16
	Ceftazidime	>64	1 to >64	34
	Ceftazidime-avibactam	>64	1 to >64	66
	Colistin	8	1 to > 8	75
	Aztreonam	32	2 to >32	9
	Amikacin	64	<4 to >64	69
	Ciprofloxacin	>4	<0.25 to >4	9
	Cefepime	>16	2 to >16	16
	Tigecycline	>4	1 to >4	NA
S. maltophilia (*n* = 50)	Cefiderocol	0.25	<0.03 to 4	100
	Ceftolozane-tazobactam	>64	0.5 to >64	NA
	Meropenem	>64	1 to >64	NA
	Ceftazidime	>64	2 to >64	38
	Ceftazidime-avibactam	>64	1 to >64	NA
	Colistin	>8	<0.5 to >8	NA
	Trimethoprim-sulfamethoxazole	0.5/9.5	<0.03/0.57 to 2/38	98
	Amikacin	>64	<4 to >64	NA
	Ciprofloxacin	>4	0.5 to >4	NA
	Cefepime	>16	8 to >16	NA
	Tigecycline	>4	<0.25 to >4	NA

Overall, 466 (97.5%) of the 478 isolates were susceptible to CFDC. Selected susceptibility test results are shown in Table 1. Against 52 extended-spectrum β-lactamase (ESBL)-positive E. coli isolates, CFDC had an MIC_90_ value of 2.0 mg/liter (range, <0.03 to 4.0 mg/liter). Comparator agents active against these isolates included amikacin, ceftazidime-avibactam, ceftolozane-tazobactam, meropenem, and tigecycline. Against 37 ESBL-positive K. pneumoniae isolates, CFDC had an MIC_90_ value of 2.0 mg/liter. Overall, 36 (97%) of 37 isolates were susceptible to CFDC, with a lone isolate having an MIC of >64.0 mg/liter. Among comparator agents, amikacin, ceftazidime-avibactam, and meropenem had appreciable activity against these isolates.

## 

### Activity against carbapenem-resistant *Enterobacteriaceae*.

Forty carbapenem-resistant *Enterobacteriaceae* (CRE) (23 K. pneumoniae, 10 E. coli, and 7 Enterobacter cloacae) were tested. The MIC_90_ of CFDC against these isolates was 4.0 mg/liter, with 37 (92.5%) of the 40 isolates having CFDC MICs of ≤4.0 mg/liter. Three isolates (7.5%) had CFDC MICs of >4 mg/liter, including 2 *Klebsiella* and one *Enterobacter* species isolates. Among comparator agents, only tigecycline was active against these organisms, with an MIC_90_ of 4.0 mg/liter.

### Activity against other *Enterobacteriaceae*.

Cefiderocol had good activity against ESBL-negative E. coli and *Klebsiella* spp. and against *Citrobacter* spp. and *Serratia* spp. (data not shown). Among comparator agents, amikacin, ceftazidime-avibactam, ceftolozane-tazobactam, meropenem, and tigecycline were active against these isolates. Most agents were less active against E. cloacae than they were against *Citrobacter* spp. While CFDC inhibited 34 (89%) of 38 *Enterobacter* species isolates at ≤4.0 mg/liter, 2 isolates had MICs of 8.0 mg/liter and 2 had MICs of >64.0 mg/liter. All agents except colistin had good activity against *Serratia* spp.

### Activity against nonfermenting Gram-negative bacilli.

CFDC was the most active agent tested against S. maltophilia isolates, with an MIC_90_ of 0.25 mg/liter and a range of <0.03 to 4.0 mg/liter. Among comparators, only trimethoprim-sulfamethoxazole was active against S. maltophilia isolates, CFDC was active against Acinetobacter spp. isolates (MIC_90_, 4.0 mg/liter). Two of 20 isolates tested were resistant to CFDC, with MICs of 16.0 and >64.0 mg/liter, respectively. Among comparator agents, amikacin, colistin, and tigecycline inhibited ≥90% of isolates. The MIC_90_ of CFDC against 15 isolates of *Achromobacter* spp. was 0.125 mg/liter. Among comparator agents, imipenem had the best activity.

CFDC inhibited all 38 P. aeruginosa isolates that did not exhibit multidrug resistance (MDR) at ≤1.0 mg/liter. Comparator agents with activity against these isolates included ceftolozane-tazobactam, ceftazidime-avibactam, amikacin, colistin, and ceftazidime. Against 32 MDR P. aeruginosa isolates, CFDC was the most active agent tested, with an MIC_90_ of 1.0 mg/liter. Only 1 isolate was resistant to CFDC, with an MIC of >64.0 mg/liter. The activity of comparator agents against these isolates was uniformly poor.

### Activity against uncommon organisms.

Cefiderocol inhibited all 7 Burkholderia cepacia isolates at ≤0.25 mg/liter, all 7 *Pantoea* spp. isolates at ≤1.0 mg/liter, all 7 Sphingomonas paucimobilis isolates at ≤0.5 mg/liter, and all 3 Elizabethkingia meningoseptica isolates at ≤4.0 mg/liter. One of 8 Rhizobium radiobacter isolates was nonsusceptible to CFDC (MIC, 8.0 mg/liter).

### Nonsusceptible isolates.

CFDC was associated with the lowest level of nonsusceptibility (Table 1). The highest level of nonsusceptibility to CFDC was seen among non-CRE *Enterobacter* spp. isolates, with 2 (5.3%) of 38 isolates being nonsusceptible. Many comparators had nonsusceptibility percentages of <2%. Of note, MDR P. aeruginosa nonsusceptibility to CFDC was 3.1%, whereas the nonsusceptible range for comparator agents was 25% to 91%. The MIC distributions for individual organisms and antimicrobial agents are presented in Table 2. Distributions for CFDC showed lower MICs for nonsusceptible organisms, including CRE, MDR P. aeruginosa, and S. maltophilia, than those of all other agents tested.

**TABLE 2:** *In vitro* activity of cefiderocol and comparator agents against commonly resistant bacterial isolates from cancer patients using MIC distribution data

Isolate, type	Antimicrobial agent	Distribution (*n*) at MIC (mg/liter) of:														
		<0.03	0.03	0.06	0.12	0.25	0.50	1	2	4	8	16	32	64	>64	Other
ESBL positive																
E. coli (*n* = 52)	Cefiderocol	2		2	7	3	14	13	8	3						
	Meropenem	32	2	14	3	1										
	Ceftolozane-tazobactam				6	15	14	3	3	3	1	1	1	3	2	
	Ceftazidime							3	1	4	7	12	7	8	10	
	Ceftazidime-avibactam	3		5	18	15	3	4	2	2						
	Cefepime							2	2	4	8	11				
	Aztreonam							1		3	11	10	19			
	Amikacin										14	7	1			
	Colistin							48	3	1						
	Ciprofloxacin					2			1							2 (<0.25); 47 (>4)
K. pneumoniae (*n* = 37)	Cefiderocol				8	16	8		4						1	
	Meropenem	5		14	14	1	1			1						
	Ceftolozane-tazobactam					2	7	4	10	8	1		2		3	
	Ceftazidime									1	1	3	8	8	16	
	Ceftazidime-avibactam	1		1	3	15	13	3							1	
	Cefepime							1	3		4	5				24 (>16)
	Aztreonam										1	6	22			8 (>32)
	Amikacin										6	4			2	25 (<4)
	Colistin							23	8	1						5 (>8)
	Ciprofloxacin							1	5	2						4 (<0.25); 25 (>4)
	Tigecycline						4	3	6	7						10 (<0.25); 7 (>4)
E. cloacae (*n* = 38)	Cefiderocol	1		1	1	3	9	8	8	3	2				2	
	Meropenem	10		8	6	6	4	0	1	1	1			1		
	Ceftolozane-tazobactam			1	6	7	3	1	3	1	5	2	2	1	6	
	Ceftazidime					9	4	1			1	3	3		19	
	Ceftazidime-avibactam				4	12	6	8	3	3		16				
	Cefepime							1	2	2	1	2				22 (<0.5); 8 (>!6)
	Aztreonam							3	1	3		2	9			10 (<0.5); 10 (>32)
	Amikacin										3	3				32 (<4)
	Colistin							18	4	1	1					14 (>8)
	Ciprofloxacin							2	4							24 (<0.5); 8 (>4)
	Tigecycline						6	4	3	2						21 (<0.5); 2 (>4)
CRE																
E. coli (*n* = 10)	Cefiderocol			2		2		3	2		1					
	Meropenem								1	2	4	1		1	1	
	Ceftolozane-tazobactam								1	1	1	1	1	1	4	
	Ceftazidime											2		2	6	
	Ceftazidime-avibactam					1	1	3	1		2				2	
	Cefepime										1					9 (>16)
	Aztreonam											2	8			
	Amikacin											2	1	1	2	4 (<4)
	Colistin							7	1							2 (<0.5)
	Ciprofloxacin															10 (>4)
	Tigecycline						1	2	1	2						4 (<0.25)
K. pneumoniae (*n* = 23)	Cefiderocol			1		3	4	5	5	4					1	
	Meropenem	2					1			2	3		5	2	8	
	Ceftolozane-tazobactam						2	1	1				6	3	10	
	Ceftazidime												1	2	19	
	Ceftazidime-avibactam			1	2	3	5	2	2	2	1	1			4	
	Cefepime															23 (>16)
	Aztreonam											1	8			13 (>32)
	Amikacin										6	1	4	1	2	9 (<4)
	Colistin							16	1		2					4 (>8)
	Ciprofloxacin								1							22 (>4)
	Tigecycline						1	5	5	8						3 (<0.25); 1 (>4)
E. cloacae (*n* = 7)	Cefiderocol				1	1		2	1	2						
	Meropenem			2	1	1			1					2		
	Ceftolozane-tazobactam							1	2	2					2	
	Ceftazidime											1	2		4	
	Ceftazidime-avibactam					1	4								2	
	Cefepime							1		1		1			1	3 (<0.5)
	Aztreonam											2	2			3 (>32)
	Amikacin															6 (<4)
	Colistin							3	1							3 (>8)
	Ciprofloxacin															6 (<0.25); 1 (>4)
	Tigecycline							1	3	1						2 (<0.25)
P. aeruginosa, MDR (*n* = 32)	Cefiderocol	6		9	6	6		4							1	
	Meropenem						1	1	3		9	9	5		4	
	Ceftolozane-tazobactam						5	7	7	2	2	2			7	
	Ceftazidime							2		6		2	6	8	8	
	Ceftazidime-avibactam							5	2	9	5	3	4		4	
	Cefepime								2	2	1	15				12 (>16)
	Aztreonam								1	2		14	13			2 (>32)
	Amikacin										7	3	2	6	2	12 (<4)
	Colistin							3	21	4	2					2 (>8)
	Ciprofloxacin						1	2	6	3						3 (<0.25); 17 (>4)
	Tigecycline							1	1							30 (>4)
Acinetobacter spp. (*n* = 20)	Cefiderocol	8		4	1	2	1	1		1		1			1	
	Meropenem	1		1	2	8	3				1		1		3	
	Ceftolozane-tazobactam	8		1	2		1	1	3			1			3	
	Ceftazidime								4	3	2	3	3	2	3	
	Ceftazidime-avibactam			1	1		1	2	3	4	3	2	1		2	
	Cefepime							7	3	1	2	1				2 (<0.5); 4 (>16)
	Aztreonam										1	10	9			
	Amikacin										1	2			1	16 (<4)
	Colistin							9	11							
	Ciprofloxacin						1									13 (<0.5); 6 (>4)
	Tigecycline						1	4	5	1						9 (<0.25)

Illumina MiSeq short-read whole-genome sequencing was performed for the CFDC-resistant isolates, followed by an analysis focused on the presence of β-lactamase-encoding genes and the composition of major porins known to contribute to β-lactam resistance. (9) *Klebsiella* spp. isolates demonstrated outer membrane porin OmpK36, OmpK37, and OmpK35 disruption and the presence of various β-lactamases. The *Enterobacter* spp. isolates had alterations in OmpC and OmpF and the presence of AmpC and ESBLs. Finally, Acinetobacter spp. isolates had carbapenemases and various β-lactamases. No clear mechanisms for CFDC resistance were found.

The standard of care for the treatment of febrile episodes in cancer patients is prompt administration of empirical antibiotic therapy (10). GNOs are now the predominant bacterial pathogens in this setting, and resistance among many GNOs is increasing. CFDC has potent *in vitro* activity against various GNOs isolated from patients with cancer, including carbapenem-resistant organisms and MDR nonlactose fermenting organisms, including S. maltophilia. Based on these *in vitro* findings and the general exclusion of patients with cancer from registration studies, we believe future study of the clinical utility of CFDC in patients with cancer is warranted.
